# Sprint interval training in the postpartum period maintains the enhanced cardiac output of pregnancy: A case study

**DOI:** 10.1113/EP091994

**Published:** 2024-07-05

**Authors:** Normand Richard, Victoria Claydon, Michael Koehle, Anita Coté

**Affiliations:** ^1^ Biomedical Physiology and Kinesiology Simon Fraser University Burnaby BC Canada; ^2^ Richard Physiological Services Port Moody BC Canada; ^3^ School of Kinesiology University of British Columbia Vancouver BC Canada; ^4^ Division of Sports Medicine University of British Columbia Vancouver BC Canada; ^5^ School of Human Kinetics Trinity Western University Langley BC Canada

**Keywords:** athlete, blood volume, exercise, pregnancy, puerperium

## Abstract

During pregnancy an increased cardiac output ($\dot{Q}$) and blood volume (BV) occur to support fetal growth. Increased $\dot{Q}$ and BV also occur during chronic endurance exercise training and benefit performance. We investigated if sprint interval training (SIT) undertaken early postpartum maintains the elevated $\dot{Q}$ and BV of pregnancy and benefits performance. The participant, a competitive field hockey player and former cyclist, visited our laboratory at 2 weeks of gestation (baseline) and postpartum pre‐, mid‐ and post‐intervention (PP_pre_, PP_mid_ and PP_post_). Delivery was uncomplicated and she felt ready to start the SIT programme 5 weeks postpartum. Inert gas rebreathing was used to measure peak exercise $\dot{Q}$ ($\dot{Q}$_peak_); ${\dot{V}}_{O_{2}\mathrm{peak}}$ was measured with a metabolic cart; and postpartum haematological values were measured with carbon monoxide rebreathing. The 18 SIT sessions progressed from four to eight sprints at 130% of ${\dot{V}}_{O_{2}\mathrm{peak}}$ peak power output. $\dot{Q}$_peak_ increased from baseline at all postpartum time points (baseline 16.2 vs. 17.5, 16.8 and 17.2 L/min at PP_pre_, PP_mid_ and PP_post_, respectively). Relative ${\dot{V}}_{O_{2}\mathrm{peak}}$ remained below baseline values at all postpartum measurements (baseline 44.9 vs. 41.0, 42.3 and 42.5 mL/kg/min at PP_pre_, PP_mid_ and PP_post_, respectively) whereas absolute ${\dot{V}}_{O_{2}\mathrm{peak}}$ rapidly reached baseline values postpartum (baseline 3.19 vs. 3.12, 3.23 and 3.18 L/min at PP_pre_, PP_mid_ and PP_post_, respectively). Postpartum BV (5257, 4271 and 5214 mL at PP_pre_, PP_mid_ and PP_post_, respectively) and Hb_mass_ (654, 525 and 641 g at PP_pre_, PP_mid_ and PP_post_, respectively) were similar between PP_pre_ and PP_post_ but decreased alongside $\dot{Q}$_peak_ at PP_mid_. Peak power was returned to pre‐pregnancy values by intervention end (302 vs. 303 W, baseline vs. PP_post_). These findings show that SIT undertaken early postpartum defends the elevated $\dot{Q}$_peak_ of pregnancy and rapidly returns absolute ${\dot{V}}_{O_{2}\mathrm{peak}}$ and peak power to baseline levels.

## Abstract (highlights)

- **What is the central question of this study?**
  Can the enhanced cardiac output of pregnancy be maintained with strenous exercise training undertaken early postpartum.
- **What is the main finding and its importance?**
  Baseline values of absolute oxygen consumption, peak power output and peak exercise cardiac output can be regained rapidly or surpassed following 6 weeks of sprint interval training postpartum. Sprint interval training can be used by endurance trained females to safely resume training early postpartum, allowing a rapid and efficient return to baseline fitness levels.

## 1 INTRODUCTION

In 2022, more than 350,000 live births occurred in Canada (Statistics Canada, 2023). The *2019 Canadian Guideline for Physical Activity Throughout Pregnancy* was derived from evidence supporting maternal and newborn safety and health (Mottola et al., 2018). In the postpartum period, return to physical activity is encouraged when physically and medically safe, typically considered to be after 6 weeks postpartum in the absence of medical complications (Jackson et al., 2022).

Currently *performance‐focused* postpartum studies are scarce and female athletes wishing to return to activity face a lack of objective evidence. One study followed international‐level Norwegian athletes undertaking either moderate or vigorous intensity training during pregnancy, which they resumed from 6 to 12 weeks postpartum (Kardel, 2005). Following the postpartum training, the ${\dot{V}}_{O_{2}\mathrm{peak}}$ of the vigorous intensity group was greater than both baseline and that of the moderate intensity group. This finding confirmed that trained females can tolerate strenuous postpartum training and that intensity appears necessary to improve ${\dot{V}}_{O_{2}\mathrm{peak}}$. Since then, little has been done to further our knowledge of performance‐focused postpartum training. Insightful case reports include the training characteristics of the world's most successful cross‐country skier during pregnancy and postpartum (Solli & Sandbakk, 2018); a female runner who qualified for the 1992 US Olympic marathon trials 16 weeks postpartum (Potteiger et al., 1993); and training continuation during pregnancy and postpartum in a competitive cyclist (Almquist et al., 2022). In addition to aerobic power, Almquist et al., also directly measured blood volume (BV) and haemoglobin mass (Hb_mass_) providing insight into oxygen carrying capacity during and after pregnancy (Almquist et al. 2022) Hb_mass_ was higher at 1 week postpartum compared to weeks 6 and 12 postpartum, and by weeks 6 and 12, Hb_mass_ remained elevated compared to week 2 of pregnancy. This finding suggests that in the postpartum period, a positive haematological advantage persists.

Pregnancy imposes considerable changes to the body that include an increased cardiac output ($\dot{Q}$) and BV to support fetal growth (Blackburn, 2018). Measuring $\dot{Q}$ may provide further insight into the physiological adaptations of pregnancy that might influence the postpartum training response. Increased $\dot{Q}$ and BV are observed following chronic endurance exercise training; they benefit performance (Joyner & Coyle, 2008) and centrally ${\dot{V}}_{O_{2}\max}$ is influenced by $\dot{Q}$ and Hb_mass_. We are only aware of one report examining $\dot{Q}$ during exercise postpartum; however, the measurement was limited to 85% of maximum (Sady et al., 1990). Combining measures of peak exercise $\dot{Q}$ ($\dot{Q}$_peak_) and Hb_mass_ could assess the heart's pumping capacity following the hemodynamic stress of pregnancy and determine if structured training undertaken early postpartum could maintain the elevated $\dot{Q}$ and BV of pregnancy. To date, analysis of intense exercise on $\dot{Q}$_peak_ postpartum remains unexplored and is needed to understand the central contribution to ${\dot{V}}_{O_{2}\max}$ in postpartum exercise. As a first step to evaluating these responses to vigorous exercise postpartum, feasibility of such a study must initially be established. As such, this case report details the longitudinal performance and cardiovascular changes following a sprint interval training (SIT) exercise programme undertaken in the postpartum period.

## 2 METHODS

### 2.1 Ethical approval

Written informed consent was provided. Ethical approval was received from Trinity Western and Simon Fraser Universities (22G18, 30001393). This study conformed to the latest version of the *Declaration of Helsinki*, except for registration in a database.

We sought to recruit a female athlete who intended on becoming pregnant in the near future to evaluate postpartum physiology and utilize pre‐pregnancy values as baseline. The participant was asked to start the structured training when she felt ready postpartum and after receiving clearance from a physician. Training started following 1 week of exercise (>150 min) completed independently, including three trainer sessions of 30 min to ensure tolerance to the training frequency and modality, and mitigate drop‐out and injury.

**FIGURE 1:**
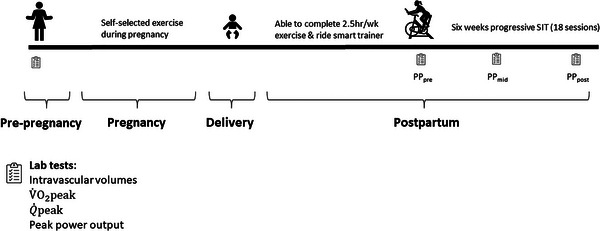
Overall study chronology. ${\dot{Q}}_{\mathrm{peak}}$, peak exercise cardiac output; SIT, sprint interval training; ${\dot{V}}_{O_{2}\mathrm{peak}}$, peak rate of oxygen consumption.

Laboratory visits were conducted at approximately 2 weeks pregnant (baseline), and on three occasions postpartum (pre‐intervention, PP_pre_; mid‐intervention, PP_mid_; post‐intervention, PP_post_). Figure 1 outlines the study chronology. The following test battery was completed at each visit. A graded exercise test (GXT) was performed to volitional exhaustion on a cycle ergometer (Velotron, Quarq Technology, Spearfish, SD, USA) with exhaled gases measured with a metabolic cart (TrueOne 2400, ParvoMedics, Murray, UT, USA) to determine ${\dot{V}}_{O_{2}\mathrm{peak}}$. Data were reported as ${\dot{V}}_{O_{2}\mathrm{peak}}$ as we did not perform post‐test supramaximal verification trials (Poole & Jones, 2017). Strong verbal encouragement was provided. The GXT began at 75 W with the load increasing by 0.37 W per second. Peak power output was the value achieved at test end. The GXT was considered maximal if respiratory exchange ratio was >1.1, heart rate (HR) was within 10 beats of predicted max HR, or ${\dot{V}}_{O_{2}\mathrm{peak}}$ plateaued despite an increasing workload. Inert gas rebreathing (Innocor, Cosmed, Italy) was used to determine ${\dot{Q}}_{\mathrm{peak}}$ (L/min) (Fontana et al., 2009; Fontana et al., 2010). Our ${\dot{Q}}_{\mathrm{peak}}$ protocol is based on the work of Bostad et al. (2023). The participant cycled at 100 W for 3 min, and then the resistance of the ergometer was increased to 95% of the peak power achieved in the GXT for 3 min. Strong verbal encouragement was provided with the goal of conducting the measurement after 2–2.5 min of work.

Hb_mass_ and plasma volume (PV) were measured with a modified version of the optimized carbon monoxide (CO) rebreathing (oCO‐rebreathe) method (Schmidt & Prommer, 2005). The participant was instructed to be well hydrated before the test. After 15 min of sitting rest, an antecubital vein sample was collected to determine haematocrit (HCT), haemoglobin concentration ([Hb]) and COHb pre‐test with a rapid blood gas analyser (Prime Plus, Nova Biomedical, Waltham, MA, USA); samples were analysed in triplicate. The participant then completed the exercise tasks. After 30 min, to allow ventilation to return to baseline, a bolus of 0.8 mL/kg of CO (99.5% purity, (Messer, Missisauga, ON, Canada) was inhaled and held for 10 s, then rebreathed in a closed system for 110 s. An oxygen‐filled bag was attached to the system, and soda lime scrubbed the CO_2_. Venous samples were taken 7 min post‐rebreathe to determine COHb post‐test. A portable CO analyser measured the remaining CO in the lungs at 4 min post‐test and in the closed loop system (Dräger Pac6500, Lübeck, Germany). Hb_mass_ and intravascular volumes were calculated (Schmidt & Prommer, 2005). Articles detailing the composition of human breast milk do not indicate the presence of red blood cells or hemoglobin as a constituent. As such, it is unlikely that breastfeeding could expose an infant to significant exogenous COHb (Ballard & Morrow, 2013; Dror & Allen, 2018; Kim & Yi, 2020). Seated blood pressure was measured after 15 min rest, immediately post and at 3 and 7 min post ${\dot{V}}_{O_{2}\max}$ test with an automated cuff (UA‐767 Plus, A&D Medical, Missisauga, ON, Canada).

The participant then completed a progressive 6‐week home‐based SIT programme of three weekly sessions on a smart trainer (Directo XR, Elite, Fontaniva, Italy) interspaced with moderate exercise of choice (Supporting information, Appendix 1). The SIT was completed on the virtual cycling platform Zwift and sessions uploaded. After each session the lead researcher inspected the power and HR tracing to confirm adherence. The SIT workload was 130% of the power achieved in the GXT. Sessions started at 20 min and four 30 s efforts and progressed to 30 min and eight 30 s efforts by week 6. Nutrition and hydration guidance were provided (Supporting information, Appendix 1). From pre‐pregnancy to intervention end, the participant supplemented with 300 mg of ferrous fumarate (Palafer, Bausch Health, Laval Québec). Estimated daily sleep, exercise energy expenditure and steps were recorded using a wrist‐worn smart‐device (Garmin Vivosmart 5 and uploaded to a research platform (Labfront, Kiipo Co., Boston, MA, USA).

## 3 RESULTS

**FIGURE 2:**
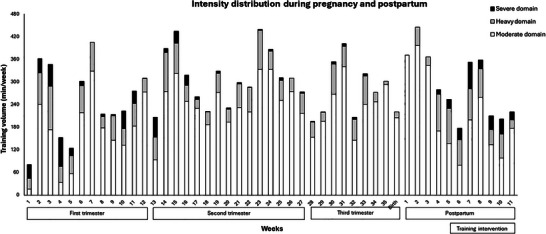
First trimester modalities: field hockey, running, cycling. Second and third trimester modality: cycling, power walking. Postpartum modalities: power walking, hiking, fitness class and SIT.

The participant was a 35‐year‐old university‐educated female, and this was her second child. She was considered Tier 3 Highly Trained in field hockey, and Tier 2 Trained in road cycling (McKay et al., 2022). Exercise intensity distribution from conception to study end is shown in Figure 2.

### 3.1 Medical outcomes

The participant experienced placenta previa during the second trimester. She modified her activity to a non‐impact modality to maintain regular exercise (cycling commuting). The placenta previa self‐resolved by mid third trimester. A term baby of 3.18 kg was delivered vaginally with vacuum assist at 37 weeks. The infant was able to immediately breastfeed after delivery and continued to do so throughout the research intervention. An episiotomy was performed and was healed by 4 weeks postpartum. Blood loss was estimated to be <500 mL at delivery.

### 3.2 Return to exercise

The participant was active until delivery. She engaged in low intensity walking during the first week postpartum. From postpartum week 2 to 3, the participant hiked and power walked. On week 4 and 5, she added postpartum exercise classes and rode her smart trainer, in addition to hiking and power walking. The participant reported feeling ready to begin structured training at 4 weeks and received medical clearance in the fifth week. She visited our laboratory for measurements the next day.

### 3.3 SIT programme

The participant increased the number of sprints from four to five after two sessions and requested a power increase to 140% of peak power after six sessions. One session mid‐intervention was replaced with laboratory performance testing. The participant completed 16/18 sessions. Missed sessions resulted from a bout of illness in week 5; SIT power was returned to 130% for the remaining sessions. No adverse events were reported. The participant thought the training was feasible, enjoyed having a personal goal, and appreciated the accountability of the training sessions. During alternative days the participant hiked or completed postpartum fitness classes. Sleep, estimated using the wearable device, was poor on 24 and fair on 18 nights of the study duration. Step count average for the study duration was 11548 steps per day and activity energy expenditure 598 kJ per day.

Cardiovascular, performance and haematological results are shown in Table 1.

**TABLE 1:** Cardiovascular, performance, and hematological results.

	Pre‐pregnancy (baseline)	PP_pre_ (5 weeks postpartum)	PP_mid_ (8 weeks postpartum)	PP_post_ (11 weeks postpartum)
Weight (kg)	70.9	76.1	76.4	74.9
BSA	1.81	1.88	1.88	1.86
Resting systolic BP (mmHg)^a^	90^b^	86	98	96
Resting diastolic BP (mmHg)^a^	60^b^	52	63	68
HCT (%)	38	41	41	40
[Hb] (g/dL)	12.7	13.7	13.5	13.5
BV (mL)	n/a	5257	4271	5214
PV (mL)	n/a	3296	2677	3316
Hb_mass_ (g)	n/a	654	525	641
Peak O_2_ uptake (mL/kg/min)	44.9	41.0	42.3	42.5
Peak O_2_ uptake (L/min)	3.19	3.12	3.23	3.18
PPO (W/kg)	4.3	3.8	4.0	4.0
PPO (W)	302	288	302	303
Peak cardiac output (L/min)	16.2	17.5	16.8	17.2
Peak cardiac index (L/min/m^2^)	8.9	9.3	8.9	9.2
Peak stroke volume (mL)	95	104	95.5	98
Peak stroke volume index (mL/m^2^)	52.38	55.35	50.73	52.73

^a^ Seated.

^b^ Taken at week 4 of pregnancy by midwife. Abbreviations: BP, blood pressure; BSA, body surface area; BV, blood volume; Hb_mass_, haemoglobin mass; HCT, haematocrit; [Hb], haemoglobin concentration; PPO, peak power output; PV, plasma volume.

### 3.4 Aerobic power and cardiac output

**FIGURE 3:**
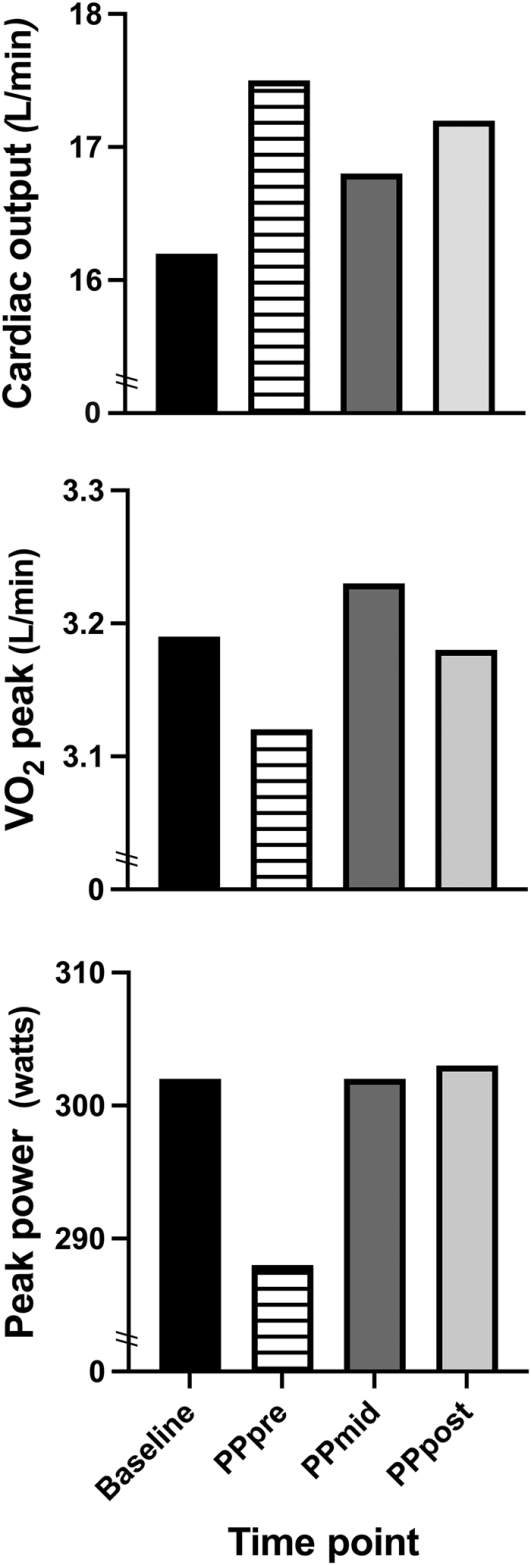
Cardiovascular and performance markers from baseline to end‐intervention.

${\dot{Q}}_{\mathrm{peak}}$ increased from baseline to PP_pre_, decreased at PP_mid_ and increased again by PP_post_. Compared to baseline, ${\dot{Q}}_{\mathrm{peak}}$ was 7.4%, 3.6% and 5.8% greater at the PP_pre_, PP_mid_, and PP_post_ time point, respectively. In all ${\dot{V}}_{O_{2}\mathrm{peak}}$ tests the participant reached volitional fatigue. Relative ${\dot{V}}_{O_{2}\mathrm{peak}}$ was decreased from baseline at all time points postpartum, whereas absolute values were equivalent to baseline after the postpartum intervention. Absolute ${\dot{V}}_{O_{2}\mathrm{peak}}$ differed from baseline by −2.2%, 1.2% and −0.3% at the PP_pre_, PP_mid_, and PP_post_ time points, respectively, whereas, relative ${\dot{V}}_{O_{2}\mathrm{peak}}$ was −9.5%, −6.1% and −5.6% lower than baseline at the PP_pre_, PP_mid_, and PP_post_ time points, respectively. Peak power was decreased at PP_pre_ by −4.9%, from baseline but returned to baseline values at mid and end intervention. Figure 3 shows the cardiovascular and performance indices from baseline to PP_post_.

### 3.5 Haematology

Haematocrit and [Hb] increased from baseline at all postpartum time points. BV, PV and Hb_mass_ were quite similar between the PP_pre_ and PP_post_ measurement, although they decreased at PP_mid_. Direct blood volume measures were unavailable at baseline due to a CO gas shortage, but HCT and [Hb] were measured.

## 4 DISCUSSION

In this case report, we demonstrate that postpartum sprint interval training, in a trained female, is well tolerated. Further we show that baseline values of absolute oxygen consumption, peak power and ${\dot{Q}}_{\mathrm{peak}}$ can be regained rapidly or surpassed following 6 weeks of SIT postpartum.

### 4.1 Maximal oxygen uptake

Absolute ${\dot{V}}_{O_{2}\mathrm{peak}}$ returned to baseline values by 11 weeks postpartum demonstrating the effectiveness of our intervention. Relative ${\dot{V}}_{O_{2}\mathrm{peak}}$ was marginally lower than baseline at PP_post_ because of the increase in body mass of pregnancy, which typically takes several months to return to baseline. PP_pre_ was measured at 5 weeks post‐delivery, where women may still retain half of their pregnancy weight (Landon et al., 2021). Lastly our participant chose to breastfeed her child. Increased breast size and the effects of breastfeeding on extravascular fluid compartments were not quantified and could influence body mass. Breast tissue can increase by ∼211 ± 16 mL from pre‐conception to 1 month postpartum, and 24 h milk production in the first 6 months averages 459 ± 48 g per breast (Cox et al., 1999; Kent et al., 1999).

There are limited data on ${\dot{V}}_{O_{2}\max}$ improvement postpartum. Almquist *et al*. followed a cyclist during pregnancy and 12 weeks postpartum. The cyclist's postpartum training appears self‐selected and predominantly of moderate intensity (Almquist et al., 2022). While absolute and relative ${\dot{V}}_{O_{2}\max}$ were measured, both remained below baseline levels (2 weeks pregnant) whereas our participant's absolute ${\dot{V}}_{O_{2}\max}$ was greater than baseline at PP_mid_, and equivalent at PP_post_. In a case study of the world's most successful female cross‐country skier, her measured absolute and relative ${\dot{V}}_{O_{2}}$, 4.0 L/min and 59.3 mL/kg/min, respectively, at lactate threshold were the same as pre‐pregnancy by 13 weeks postpartum. While most training was at moderate intensity, this skier included several ≥15 h training weeks (Solli & Sandbakk, 2018). In the marathoner case study, her ${\dot{V}}_{O_{2}\max}$ postpartum increased from week 4 to week 16 (52.2 vs. 55.3 mL/kg/min). This athlete ran 64–84 km/week over seven sessions postpartum, which included one long run, an interval session, with the remaining distance distributed over short runs. The participant also experienced a progressive fat mass loss and ultimately ran a 2:43:37 marathon (Potteiger et al., 1993).

One study followed females wishing to become pregnant and a non‐pregnant control group. Both groups exercised three to six times per week; however, the experimental group had a lower duration‐intensity during pregnancy and the postpartum period (Clapp & Capeless, 1991). At 12 and 36 weeks postpartum, the pregnant group surpassed their pre‐pregnancy ${\dot{V}}_{O_{2}\max}$ and at 36 weeks, the ${\dot{V}}_{O_{2}\max}$ of the pregnant group was greater than in the control group despite lower duration‐intensity minutes. The authors proposed that pregnancy could support central ($\dot{Q}$_peak_, BV) or peripheral (angiogenesis, increase fat metabolism) adaptations benefiting exercise performance (Clapp & Capeless, 1991). Others also report that highly trained females only increase their ${\dot{V}}_{O_{2}\max}$ postpartum following vigorous but not moderate training (Kardel, 2005). Thus, from these studies and our findings, it appears that high intensity interval training or SIT effectively increases ${\dot{V}}_{O_{2}\max}$ relatively early postpartum. Of note, our participant experienced a respiratory tract infection which affected her last 2 weeks of training and the final test session. We speculate that her PP_post_ peak power and ${\dot{V}}_{O_{2}\mathrm{peak}}$ would have been greater than baseline if had she not become unwell.

### 4.2 Cardiac output

In this participant, absolute and relative ${\dot{Q}}_{\mathrm{peak}}$ was greater at PP_pre_ compared with baseline. We suspect this reflects the effect of pregnancy and not a fitness gain, as her peak power and ${\dot{V}}_{O_{2}\mathrm{peak}}$ at this time were lower than baseline. At PP_post_, ${\dot{Q}}_{\mathrm{peak}}$ remained greater than baseline, supporting our hypothesis that SIT would defend the elevated ${\dot{Q}}_{\mathrm{peak}}$ of pregnancy. The inter‐session coefficient of variation of the Innocor is 7%, and therefore we believe our measurements fall within the device's capabilities (Fontana et al., 2009). We acknowledge the Innocor underestimates ${\dot{Q}}_{\mathrm{peak}}$ (Musch & Poole, 2024); however, as the same instrument was used for all tests we are confident in our ability to track changes within a participant over time.

It is worth considering that breastfeeding increases resting $\dot{Q}$ (Katz & Creasy, 1984; Sibolboro Mezzacappa et al., 2001). At present it is unknown if the increase in resting $\dot{Q}$ could be additive to the exercise increase in $\dot{Q}$ as we did not undertake resting measurements. However, an increased pumping ability of the heart would support exercise performance and active mothers should not be discouraged from breastfeeding. Our participant did not experience any difficulties breastfeeding, even following supramaximal efforts at 140% of peak power. Postpartum exercise trials should report breastfeeding status and any complications to develop this line of evidence further.

### 4.3 Haematology

Increased BV supports enhanced cooling and oxygen transport and so benefits exercise performance. Recreationally active females (≥5 × 30 min/week) had greater BV, PV and red cell volume than sedentary pregnant counterparts at 25 and 35 weeks gestation and 12 weeks postpartum (Pivarnik et al., 1994). Body weight was similar between groups. While the amount of exercise postpartum was unclear, PV contracts following sedentarism even in trained individuals (Coyle et al., 1986), and thus the active females were exercising enough to maintain their PV.

Our haematological measurements fall in line with those of Almquist *et al.*, who showed increased HCT, [Hb] and peak power in the postpartum period (Almquist et al., 2022). Our greatest PV and Hb_mass_ values measured at PP_pre_ are likely a carryover from pregnancy as values dropped at PP_mid_ in conjunction with a decrease in $\dot{Q}$_peak_. At the PP_post_ measurement, PV and Hb_mass_ increased again in conjunction with $\dot{Q}$_peak_. Almquist et al., similarly reported an undulating pattern in their postpartum hematological parameters (Almquist et al., 2022). Changes in erythrocytes may explain this pattern. The lifecycle of erythrocytes is decreased during pregnancy and females in late pregnancy show younger erythrocytes in their blood compared to non‐pregnant females (Lurie & Mamet, 2000). The rapid BV decrease that occurs postpartum is unlike one of a major blood loss; HCT and [Hb] are relatively stable (Lurie & Mamet, 2000). Further, erythropoiesis which occurs after delivery (Bjørke‐Monsen et al., 2012; Richter et al., 1995) could trigger the formation of new erythrocytes. A randomized, double‐blind, placebo‐controlled study has also demonstrated that at 8 weeks postpartum, HCT and erythrocyte count is higher than at any stage of pregnancy (Milman et al., 2000). In other words, the postpartum period could exhibit a plethora of erythrocytes.

Neocytolysis is a physiological mechanism which removes young erythrocytes from the circulation and has been documented in space flight and following return from altitude (Alfrey et al., 1997; Mairbäurl, 2018). Neocytolysis would regulate HCT to balance blood viscosity with oxygen carrying capacity. We propose that neocytolysis could have decreased the Hb_mass_ at the PP_mid_ time point. Although we are unaware of reports documenting this phenomenon in exercise training postpartum, neocytolysis has been previously hypothesized to occur after pregnancy (Lurie & Mamet, 2000). The increase in haematological parameters at PP_post_ is potentially related to the training effects of the exercise programme itself. It is well known that exercise training increases both PV and Hb_mass_ (Convertino et al., 1980; Montero & Lundby, 2018; Sawka et al., 2000). Recently, using the same oCO‐rebreathe technique as us, others have shown an increase in Hb_mass_, BV and $\dot{Q}$_peak_ (same device) following three SIT sessions per week for 6 weeks in both males and females (Mandić et al., 2022). Further work is needed to either refute or support the above proposed mechanisms and to confirm if a peaks and valley pattern in hematological parameters is a common response to exercise training postpartum or simply an artifact.

### 4.4 Limitations and considerations

One limitation to our observations is the absence of baseline haematological data. This limits our ability to attribute the exact role of BV expansion and/or decay on our participant's postpartum performance. We also did not perform post‐${\dot{V}}_{O_{2}\max}$ verification trials, and so we report our data as ${\dot{V}}_{O_{2}\mathrm{peak}}$. Additionally, our baseline measurement was undertaken at ∼2 weeks of gestation and not pre‐pregnancy. However, blood volume begins expansion at 6–8 weeks, with peak values seen at 32–34 weeks, and increases in resting cardiac output are only detectable at 5–8 weeks (Blackburn, 2018). Thus, as most cardiovascular changes begin after > 6 weeks of gestation our ‘baseline’ measurements likely reflect the non‐pregnant state more than the pregnant state. These encouraging findings are specific to one individual and cannot be generalized. Our participant was active before, during and after her first, and this second pregnancy, and was accustomed to vigorous exercise. She also had support from an experienced exercise physiologist. Our findings may or may not apply to Tier 4–5 athletes who have reached their physiological ceiling, or accurately reflect the responses in sedentary females. Participation in research studies during the postpartum period is challenging. We decreased barriers to participation by providing babysitting at the laboratory, sectioning part of our laboratory with privacy shields so the participant could breastfeed comfortably, and providing financial compensation to offset travel costs.

### 4.5 Conclusion

In this case study, we show that SIT postpartum is tolerable and rapidly returns absolute ${\dot{V}}_{O_{2}\mathrm{peak}}$ and peak power to baseline values. Further, SIT postpartum defends the elevated $\dot{Q}$ of pregnancy and yields a greater ${\dot{Q}}_{\mathrm{peak}}$ postpartum. We invite others to continue exploring this area, which will immensely benefit professional female athletes, recreationally active women and those working in arduous occupations.

## AUTHOR CONTRIBUTIONS

Study conception and design: Normand Richard, Anita Coté; data collection: Normand Richard; analysis and interpretation of results: Normand Richard, Anita Coté; draft manuscript preparation: Normand Richard, Victoria Claydon, Michael Koehle, Anita Coté. All authors have read and approved the final version of this manuscript and agree to be accountable for all aspects of the work in ensuring that questions related to the accuracy or integrity of any part of the work are appropriately investigated and resolved. All persons designated as authors qualify for authorship, and all those who qualify for authorship are listed.

## CONFLICT OF INTEREST

None declared.

## Supporting information

Appendix 1. Postpartum study home instructions.

## Data Availability

All data supporting the results are in the paper itself.
