# Supplementary material for: Sprint interval training in the postpartum period maintains the enhanced cardiac output of pregnancy: A case study
Source: Exp Physiol. 2024 Jul 5;109(10):1629–36. doi: 10.1113/EP091994 (PMC11442777; doi:10.1113/EP091994)
Supplement: Supplementary file 1 — Appendix 1. Postpartum study home instructions. [file EPH-109-1629-s001.docx]

**Appendix 1.**

**Postpartum study home instructions**

**SPRINT INTERVAL SESSIONS**

Ideally do this session when you know your baby will have a good nap (30min or more) or you can have someone watch them so you can do the session uninterrupted. The goal is to do three sessions per week, interspaced with three endurance session of your choice (hiking, swimming, walking). The number of intervals per session will increase as the program progresses.

I will email you the custom Zwift workouts based on your power from our first lab visit. To upload the file (ending in .zwo) into Zwift. <https://support.zwift.com/en_us/custom-workouts-ryGOTVEPs#Sharing>


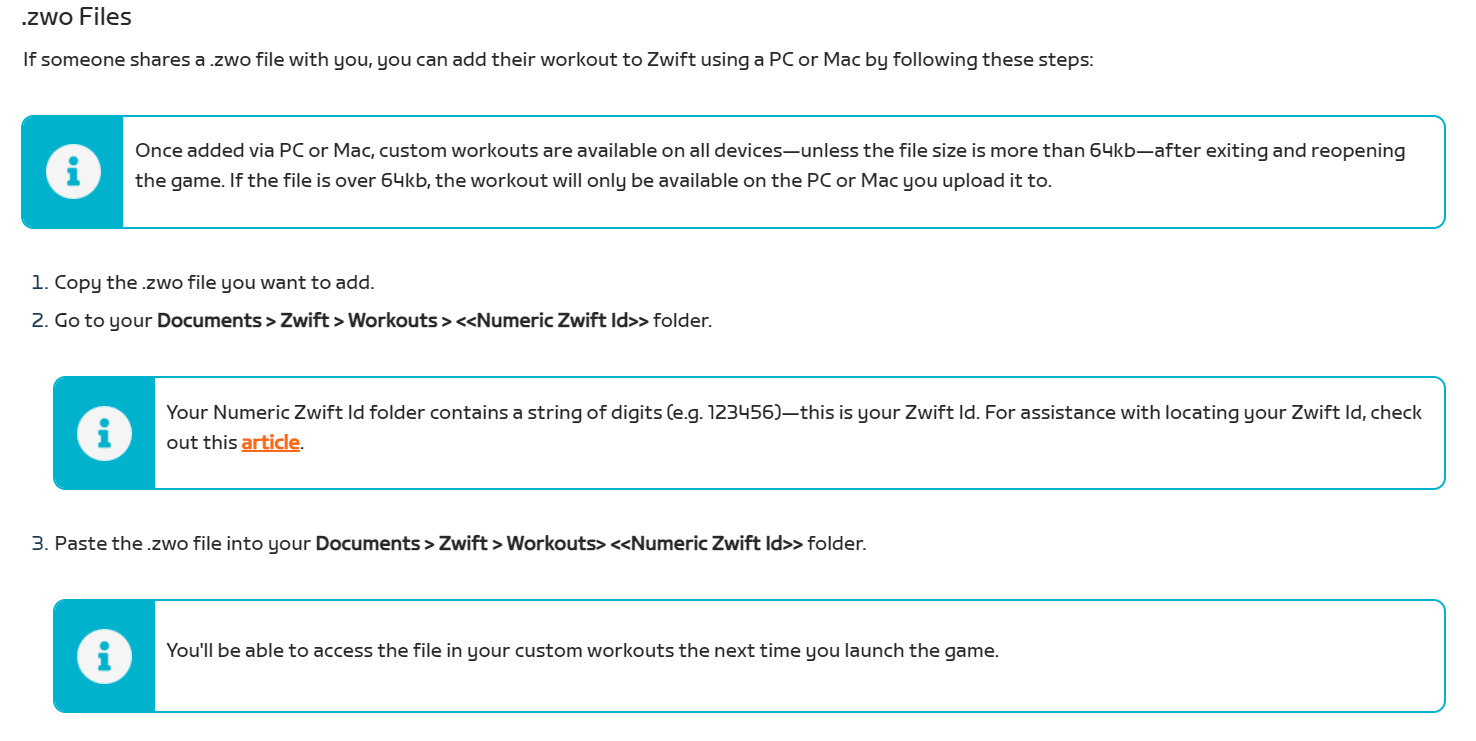


One hour before:

1. Make sure you start the session well hydrated (as indicated by pale yellow urine) and eat a light carbohydrate snack one hour beforehand (e.g., banana, granola bar, half bagel) if you have not eaten in the last three hours
2. This is good time to pump/feed if you need to

Getting ready:

1. Put on your heart rate monitor
2. Get dressed! Socks, shorts, supportive sports bra, and shirt
3. Have a bottle ready with water or sports drink
4. Have a large towel nearby, it is expected that you will sweat a lot (hence why you must start the session well hydrated and drink throughout the session, and continue drinking a normal amount afterwards)
5. It is okay to listen to music
6. You can use a fan or air conditioning

Get going

1. Calibrate your trainer and pair it to Zwift
2. Follow the prescribed warmup
3. Complete the prescribed amount of 30sec sprints **ALL-OUT**. There should be no attempt at pacing. It’s okay if your power drops. What is important is that your effort is as hard as you can. In between sprints pedal at a low intensity for 60sec.
4. Follow the prescribed cool down for 5min at ~100watts
5. Stop the workout and save the Zwift and Garmin/Wahoo file

Afterwards

1. Share the Zwift file with Normand at the end of your workout
2. Fill in the three questions on the LabFront application
3. Make sure to hydrate for the rest of the day, but no need to over do it

**If the workouts are too easy**: If you can easily do the prescribed sprints for three sessions in a row and it feels too easy contact me, I will add another sprint or increase the power.

**If the workouts are too hard:** If you struggle to finish half the workout on two occasions, let me know we’ll adjust the power.

**ENDURANCE SESSIONS**

Complete 30-45min of “cardio” type exercise of your choice at a moderate intensity (Zone 2) three times per week. This could be jogging, hiking, swimming, or cycling. Ideally record the activity with your heart rate monitor or smart watch but this is not essential. You should be able to talk but not sign a song.

**REST DAY**

Take one day off with no exercise per week.

**MISSED SESSIONS**

We understand that being a parent is difficult and that your family and health is your first priority. While we hope you can complete every session you can still stay in the study if you miss sessions. Just note it in your training calendar (including the cause if possible). You don’t need to make up for missed sessions. Please do not do more than 3 interval sessions per week.

**TRAINING CALENDAR**

| **Week** | **Day 1** | **Day 2** | **Day 3** | **Day 4** | **Day 5** | **Day 6** | **Day 7** |
| --- | --- | --- | --- | --- | --- | --- | --- |
| **1** | 4*30sec | 30min aerobic | 4*30sec | 30min aerobic | 4*30sec | 30min aerobic | Rest |
| **2** | 4*30sec | 30min aerobic | 4*30sec | 30min aerobic | 4*30sec | 30min aerobic | Rest |
| **3** | 5*30sec | 30min aerobic | 4*30sec | 30min aerobic | 5*30sec | 30min aerobic | Rest |
| **4** | 6*30sec | 30min aerobic | 5*30sec | 30min aerobic | 6*30sec | 30min aerobic | Rest |
| **5** | 2*(4*30 sec) | 30min aerobic | 6*30sec | 30min aerobic | 2*(4*30 sec) | 30min aerobic | Rest |
| **6** | 2*(4*30 sec) | 30min aerobic | 6*30sec | 30min aerobic | 2*(4*30 sec) | 30min aerobic | Rest |
| **7** | Rest | Rest | Test day |  |  |  |  |

**VISUAL SPRINT INTERVAL TRAINING WORKOUT BREAKDOWN**

The number of intervals will progress as the program unrolls

Time

**10 min warm up**

**5 min cool down**

**60 sec recovery**

**30 sec all-out**

Intensity
